# Supplementary material for: Why do Chinese migrant workers return? Exploring economic push-pull factors and emotional ties
Source: PLoS One. 2025 Jul 17;20(7):e0325848. doi: 10.1371/journal.pone.0325848 (PMC12270181; doi:10.1371/journal.pone.0325848)
Supplement: S1 Text — (PDF) [file pone.0325848.s001.pdf]

Questionnaire

Constructs	Items
**Basic Information Survey**	BIS1 – What is your gender?<br>BIS2 – What is your age?<br>BIS3 – Where is your current workplace located?<br>BIS4 – What type of work do you do?<br>BIS5 – How long have you worked at your current workplace?<br>BIS6 – What is your family situation in your hometown?
**Family Attachment**	FA1 – Do you often miss your relatives left in your hometown (e.g., parents, spouse, children)?<br>FA2 – When working in the city, do you worry about the living and health of your relatives in your hometown?<br>FA3 – Do you feel guilty or uneasy because of being separated from your relatives in your hometown?<br>FA4 – Do you think regularly visiting your relatives is very important for maintaining your relationship with your family?
**Place Emotion**	PE1 – Do you have a deep affection for the customs and traditions of your hometown?<br>PE2 – Do you often miss the lifestyle and customs of your hometown?<br>PE3 – Do you feel that the culture and traditions of your hometown have significant meaning to you?<br>PE4 – Do you think your hometown is the best place to live in your mind?<br>PE5 – Do you often miss the natural environment and familiar streets of your hometown?
**Communit Involvement**	CI1 – Do you often participate in community activities or social gatherings in your hometown?<br>CI2 – Do you participate in rural construction management?<br>CI3 – Do you maintain good relationships and interactions with your neighbors in your hometown?<br>CI4 – Do you think returning to your hometown allows you to actively participate in community affairs and activities?<br>CI5 – Do you feel that participating in community affairs in your hometown is important to you?
**Quality of Life in Hometown**	QLH1 – Do you think the medical services in your hometown can meet your needs?<br>QLH2 – Do you think the educational resources in your hometown are sufficient for your children?<br>QLH3 – Do you feel that you can enjoy enough leisure and entertainment facilities in your hometown?<br>QLH4 – Do you think the security situation in your hometown makes you feel safe?
**Perceived Benefits**	PB1 – Do you think returning to your hometown can provide you with similar or better income compared to your current job?<br>PB2 – Do you think living in your hometown can bring higher quality of life to you and your family?<br>PB3 – Do you think you can better balance work and family life in your hometown?<br>PB4 – Do you feel that returning to your hometown allows you to better take care of your family while enjoying more family time?<br>PB5 – Do you think developing in your hometown will give you more opportunities for personal growth and career development?<br>PB6 – Do you feel that returning to your hometown can provide better social support and resources?
**Perceived Trust**	PT1 – Do you trust that the local government in your hometown can provide sufficient support and help?<br>PT2 – Do you believe that the policies of your hometown can effectively promote economic development and provide employment opportunities?<br>PT3 – Do you feel that the social environment in your hometown is trustworthy and safe?<br>PT4 – Do you think the infrastructure and public services in your hometown can meet your daily living needs?
**Perceived Cost**	PC1 – Do you think it is difficult to find a stable job after returning to your hometown?<br>PC2 – Are you worried that you cannot obtain the same level of medical and educational resources as in the city after returning to your hometown?<br>PC3 – Do you think living in your hometown may lead to reduced income and lower quality of life?<br>PC4 – Do you feel that adapting and reintegrating into the community after returning to your hometown will be difficult?<br>PC5 – Do you think the cost of living in your hometown (e.g., house maintenance, agricultural investment) will increase after returning?
**Support**	ST1 – Support the development of rural tourism.<br>ST2 – Support the development of rural infrastructure.<br>ST3 – Support the development of the rural environment.<br>ST4 – Support the development of rural policies.
**Governme Support**	GS1 – Do you think the government's policies for returning home are practically helpful to you?<br>GS2 – Do you think the financial subsidies provided by the government can reduce your economic burden of returning home?<br>GS3 – Do you trust that the government can provide sufficient employment opportunities and vocational training for returning migrant workers?<br>GS4 – Do you think the government's policies can effectively improve the infrastructure and living conditions in your hometown?<br>GS5 – Do you feel that the government's publicity and policies can encourage you to be more willing to return to your hometown to work and live?
**Intention to Return Home**	IRH1 – Do you have a strong intention to return to your hometown to work and live in the near future?<br>IRH2 – Have you considered giving up job opportunities in the city to develop in your hometown?<br>IRH3 – Do you think the development potential of your hometown is enough to attract you to return to work there?<br>IRH4 – Do you plan to settle and live in your hometown long-term in the next few years?
