# Supplementary material for: Why do Chinese migrant workers return? Exploring economic push-pull factors and emotional ties
Source: PLoS One. 2025 Jul 17;20(7):e0325848. doi: 10.1371/journal.pone.0325848 (PMC12270181; doi:10.1371/journal.pone.0325848)
Supplement: Attachment — Submitted filename: Response to Reviewers-6.Mar.pdf [file pone.0325848.s003.pdf]

Dear Reviewer,

Thank you for your thoughtful and constructive feedback on our manuscript titled “Why Do Chinese Migrant Workers Return? Exploring Economic Push-Pull Factors and Emotional Ties”. This study utilizes the Push-Pull Theory from migration theory to develop a comprehensive model. This model investigates how perceived benefits, trust, costs, and both personal and government support affect migrant workers’ willingness to return. It provides novel insights into how both economic incentives and emotional ties drive migrant workers’ decisions to return, offering a more nuanced understanding of migration dynamics in China. We greatly appreciate your time and effort in reviewing our work and providing detailed comments to help us improve the paper. Below, we address each of your major comments and outline the revisions we have made in response to your suggestions. In addition, we also uploaded all the raw data from this study to a publicly available database for viewing, Mendeley Data (Reserved DOI: 10.17632/s7ttjys754.1).

We believe that the revisions made in response to your comments have significantly strengthened the manuscript. The integration of specific data, expanded theoretical discussions, and detailed methodological explanations provide a more robust foundation for our study. We are confident that these improvements will enhance the clarity, relevance, and impact of our work. Thank you once again for your valuable feedback. We look forward to your response and hope that the revised manuscript meets the high standards of PLOS ONE.

Sincerely,

Zhou Qiong
School of Public Administration, Hunan Agricultural University, Changsha, Hunan, China

Response to Reviewers' Comments :

Reviewers: 1

The authors should discuss the motivation of this study more explicitly. The authors should clarify how this study differs from previous studies and explicitly articulate its unique contributions in the introduction. For instance, how this paper differs from Bi et al. 92019) discussed the migrant workers' social networking and Fan et al. (2025) on land ownership and migrants' settlement and maximizing the labor economy. So, how is this study different from previous studies? Clearly outlining these aspects will enhance the paper's impact and contextual significance.

References:

https://doi.org/10.1016/j.habitatint.2019.02.008

https://doi.org/10.1016/j.landusepol.2024.107411

We fully acknowledge the importance of explicitly discussing the motivation behind our study and distinguishing it from previous research. To address this, we have revised the introduction to include a more detailed rationale for conducting this study. In the revised introduction, we have explicitly highlighted the gaps in the existing literature and articulated how our study contributes to the field. Unlike Bi et al. (2019), which focuses on social networking, and Fan et al. (2025), which examines land ownership and labor economy, our study integrates economic push-pull factors with emotional ties (e.g., family attachment, community involvement, and quality of life) to provide a more holistic understanding of return migration decisions. We also emphasize that our study extends the Push-Pull Theory by incorporating emotional bonds as a critical dimension, which has been underexplored in prior research on Chinese migrant workers.

The ~~"~~Push-Pull Theory~~" in migration studies was first proposed by American scholar~~, initially proposed by E.S. Lee in the 20th century, provides a foundational framework for understanding migration dynamics [2]. Lee categorized migration influences into ~~"push" and "pull" forces [2]. Push factorsare adverse conditions that~~ "push" factors, such as poverty and unemployment, which drive individuals away from their place of origin, ~~such as poverty, unemployment, and environmental degradation. In contrast, pullfactors are favorable conditions that attract individuals to new destinations, including~~ and "pull" factors, such as better job opportunities ~~,~~ and improved living conditions, ~~and enhanced social welfare. This~~ which attract individuals to new destinations. While this theory has been widely applied to various migration ~~scenarios~~contexts, including rural-to-urban ~~migration,~~ and international migration, ~~and refugee movements. However, applications of the Push-Pull Theory in studying migrant workers' return intentions remain limited. This limitation exists because most previous studies have focused on migrant workers' motivations and adaptation in urban settings. The trend of returning home has gained attention only recently, leaving a gap in research on this topic. Furthermore, China~~its application to the study of migrant workers' return intentions remains limited. Existing research, such as Bi et al. (2019), has focused on social networking among migrant workers [3], while Fan et al. (2025) has examined land ownership and labor economy dynamics [4]. However, these studies have not fully explored the interplay between economic incentives and emotional ties in shaping return migration decisions. This study addresses this gap by integrating economic push-pull factors with emotional ties, such as family attachment, community involvement, and

quality of life, to provide a more comprehensive understanding of return migration [5]. Unlike previous studies, which have primarily focused on urban adaptation and settlement, our research emphasizes the role of emotional bonds in influencing migrant workers’ ~~s unique national conditions make the transition of surplus rural labor both challenging and urgent. Studying migrant workers’ return intentions involves numerous challenges, including economic benefits, social support, cultural identity, and family responsibilities. These complexities increase the difficulty of theoretical analysis and require more rigorous empirical research.~~

~~This study constructs a comprehensive model of migrant workers’ returnintentions using the ”~~decisions to return. By constructing a model based on the Push-Pull Theory~~” as a foundation. The~~, this study aims to identify key factors influencing ~~migrant workers’ decisions to return and to examine the interactionsamong these factors [5]. In addition to classic push and pull factors, we include emotional ties, a sense of belonging, and social connections as influencing variables, which may directly impact the decision to return. Furthermore, we conducted a quantitative survey to gather empirical data. Government support is included~~ return intentions and examine their interactions. Additionally, this study includes government support as a moderating variable to assess its ~~effect on the relationshipsbetween factors. The results offer valuable insights for government agencies and relevant departments. These insights support the development of targeted policies to encourage migrant workers to return, thereby fostering~~ impact on these relationships. Government policies, such as subsidies for returning entrepreneurs, vocational training programs, and rural infrastructure investments, can amplify the economic appeal of returning home while also addressing emotional factors, such as improved quality of life and family reunification. Through a quantitative survey, this study gathers empirical data to test our model, offering valuable insights for policymakers seeking to promote rural economic growth and revitalization by encouraging migrant workers to return [6].

Reviewers: 1

While the Push-Pull Theory is well established, a more explicit discussion of how this study extends or refines the theory would strengthen its contribution. The manuscript should clearly articulate how integrating emotional bonds enhances the explanatory power of the Push-Pull framework.

We appreciate your suggestion to more explicitly discuss how our study extends or refines the Push-Pull Theory. In the revised manuscript, In the revised manuscript, we elaborated in detail in the theoretical framework section how integrating emotional bonds enhances the explanatory power of the push-pull framework. We argue that emotional ties act as both push and pull factors, influencing migrants' perceptions of economic incentives and their ultimate return decisions. This extension provides a more nuanced understanding of return migration, particularly in the context of Chinese migrant workers, where family and community ties play a significant role.

~~However, the application of the~~ Despite its broad applicability, the Push-Pull Theory ~~to understand migrant workers' return intentionsremains limited. Previous studies have primarily~~ has been underexplored in the context of migrant workers' return intentions. Existing research has predominantly focused on the motivations and adaptation processes of migrant workers ~~relocating to urban areas. The trend of migrant workers returning to their hometowns has only recently garnered attention as a new phenomenon [17]. As China continues to develop economically and urbanize~~in urban areas, with limited attention to the factors driving their decisions to return to their hometowns [17]. This gap is particularly notable given the recent emergence of return migration as a significant phenomenon in China. As the country undergoes rapid economic development and urbanization, the complexity of migrant workers~~'~~' return intentions has become increasingly ~~evident~~apparent. These intentions are ~~shaped by a variety of~~ influenced by a combination of economic, social, and emotional factors, including economic benefits, family responsibilities, cultural identity, and social support [18]. ~~Rising~~ For instance, rising urban living costs, ~~intensified~~ heightened job competition, and challenges in social integration have ~~become new "push " factors, prompting migrant workers to consider leaving cities. Conversely, development opportunities in their hometowns,~~ emerged as new push factors, discouraging migrant workers from remaining in cities. On the other hand, opportunities for local development, strong family ties, access to land, and ~~local cultural connections serve as "pull " factorsthat encourage~~ cultural connections in their hometowns act as pull factors, encouraging their return [19]. ~~Integrating the~~

Reviewers: 1

Some key concepts, such as "perceived trust" and "return support," need clearer definitions and justification in terms of measurement. Providing a detailed explanation of how these variables were operationalized would improve clarity.

Thank you for pointing out the need for clearer definitions and operationalization of key concepts such as "perceived trust" and "return support." In the revised manuscript, we have revised all professional terms for better reading.

3.1 Conceptual model

~~The~~

This study employed the Likert five-point scale to quantify the relationships among variables. Specifically, family attachment assessed the emotional bond between individuals and their hometown relatives; local emotions gauged identification and attachment to hometown culture, environment, and traditions. Community involvement was measured by participation frequency, role engagement, and social network extent. Quality of life in hometown encompassed dimensions such as healthcare, education, leisure, and public security. Perceived benefits involved expectations regarding economics, quality of life, work-family balance, and personal development. Perceived trust pertained to trust in the government, policies, and social environment, while perceived costs encompassed economic, time, and psychological burdens. Support was quantified by attitudes towards rural tourism, construction, environment, and policies. Government support was evaluated through satisfaction with return policies, subsidies, training, and infrastructure improvements. The intention to return home reflected individuals' intentions to work, live, and settle in their hometown. The conceptual model is shown as Fig. 1.

Reviewers: 1
The paper should discuss how representative the sample is of the broader population of migrant workers in China. Were specific regions, industries, or demographic groups over- or underrepresented? This is not shown in Figure 2. Addressing this would improve the generalizability of the findings.

Thank you for your valuable feedback. We appreciate your suggestion regarding the representativeness of our sample in relation to the broader population of migrant workers in China. We acknowledge that discussing potential over- or underrepresentation of specific regions, industries, or demographic groups would enhance the generalizability of our findings. We Conduct a detailed analysis of our sample composition, comparing it with national statistics on migrant worker distribution across regions, industries, and demographic groups. Moreover, we discussed the implications of our sample's representativeness for the interpretation and generalizability of our findings.

The online part distributed questionnaires through social media and email platforms, while the offline part distributed paper questionnaires in public places, communities, and within enterprises. A total of 400 questionnaires were distributed, and 312 valid questionnaires were ultimately collected, with an effective response rate of 78%. Data collection occurred from March to July 2024 across various cities in Hunan Province, China. This study implemented strict confidentiality measures during the questionnaire design phase to ensure that all respondents' personal information and answers were not associated with their identities. The data collection process may be subject to response bias, as participants who chose to respond may have stronger opinions or experiences related to return migration. Additionally, the response rate, while acceptable for this type of study, may not fully capture the diversity of perspectives among migrant workers. To mitigate these issues, we employed stratified sampling to ensure a balanced representation of key demographic and occupational groups. Furthermore, we conducted post-hoc checks to assess non-response bias, comparing early and late respondents on key variables, and found no significant differences. Hunan Province was selected as the research site due to its status as a major source of migrant workers in China. The province has a significant outflow of labor to economically developed regions, particularly the Pearl River Delta, making it a representative case for understanding return migration trends. Its socio-economic characteristics, including a mix of rural and urban areas, align with those of many central and western provinces, which are also major sources of migrant workers. This makes Hunan an ideal case study for examining the factors influencing return migration in regions with similar economic and cultural contexts.

Reviewers: 1
Lines 330-331 “Data collection occurred from March to July 2024 across various cities in Hunan Province, China, resulting in 312 valid responses...” This dataset focuses on migrant workers from Hunan. How can it be generalised to Chinese migrant workers? A qualification is required if it can be generalised. Why Hunan? Are there any dedicated reasons to select migrant workers from Hunan?

Thank you for your careful consideration and valuable suggestions. Added a detailed discussion in the methodology section (Lines 437-466) explaining why Hunan Province was selected. Hunan is a major source of migrant workers in China, with a significant outflow of labor to economically developed regions like the Pearl River Delta. Its socio-economic characteristics are representative of many central and western provinces, making it a suitable case study for understanding return migration trends.

The online part distributed questionnaires through social media and email platforms, while the offline part distributed paper questionnaires in public places, communities, and within enterprises. A total of 400 questionnaires were distributed, and 312 valid questionnaires were ultimately collected, with an effective response rate of 78%. Data collection occurred from March to July 2024 across various cities in Hunan Province, China. This study implemented strict confidentiality measures during the questionnaire design phase to ensure that all respondents’ personal information and answers were not associated with their identities. The data collection process may be subject to response bias, as participants who chose to respond may have stronger opinions or experiences related to return migration. Additionally, the response rate, while acceptable for this type of study, may not fully capture the diversity of perspectives among migrant workers. To mitigate these issues, we employed stratified sampling to ensure a balanced representation of key demographic and occupational groups. Furthermore, we conducted post-hoc checks to assess non-response bias, comparing early and late respondents on key variables, and found no significant differences. Hunan Province was selected as the research site due to its status as a major source of migrant workers in China. The province has a significant outflow of labor to economically developed regions, particularly the Pearl River Delta, making it a representative case for understanding return migration trends. Its socio-economic characteristics, including a mix of rural and urban areas, align with those of many central and western provinces, which are also major sources of migrant workers. This makes Hunan an ideal case study for examining the factors influencing return migration in regions with similar economic and cultural contexts.

Reviewers: 1
Figure 2 is interesting. Why do migrant workers prefer cities like Guangzhou and Shenzhen over Beijing and Shanghai? Is it common for Hunan workers not to go to the largest two Chinese cities?

You noted the interesting pattern in Figure 2, where migrant workers from Hunan prefer cities like Guangzhou and Shenzhen over Beijing and Shanghai. We have expanded the discussion of this finding in the results section (Lines 459-470) to provide context:

Guangzhou and Shenzhen, located in the Pearl River Delta, are geographically closer to Hunan and have historically attracted many migrant workers from the province due to their booming manufacturing and service sectors. In contrast, Beijing, and Shanghai, while economically dominant, are farther away and have higher living costs, which may deter some migrant workers. This pattern aligns with broader migration trends in China, where proximity and economic opportunities play a significant role in destination choices. The population mainly flows into Guangzhou and Shenzhen areas, as shown in Figures.

(source: https://www.hunan.gov.cn/hnszf/hnyw/bmdt/202107/t20210718_19944083.html.)

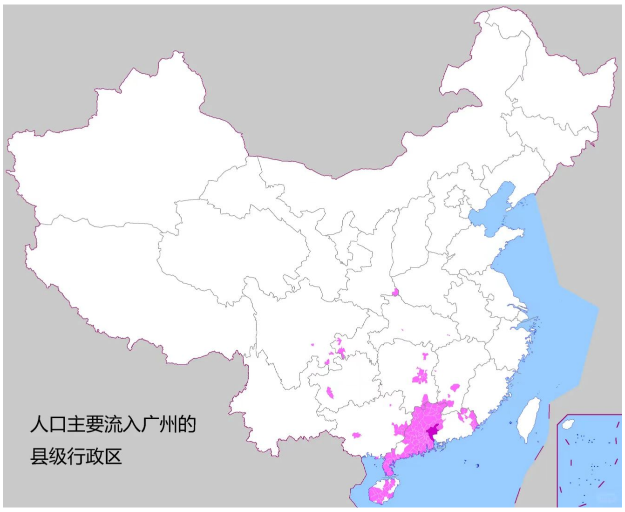

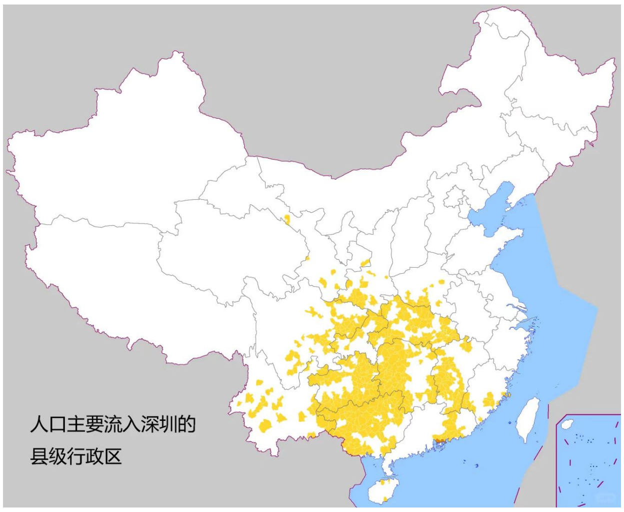

and 17 were 56 or older, representing smaller age groups. In terms of workplace location, Shenzhen had the largest number of respondents (86), followed by Guangzhou (72), Chengdu (69), Hangzhou (62), and Shanghai (16). The smallest group was in Beijing, with only 7 respondents. The distribution suggests that migrant workers are primarily concentrated in the Pearl River and Yangtze River Delta regions, especially in cities like Shenzhen and Guangzhou. Guangzhou and Shenzhen, located in the Pearl River Delta, are geographically closer to Hunan and have historically attracted many migrant workers from the province due to their booming manufacturing and service sectors. In contrast, Beijing, and Shanghai, while economically dominant, are farther away and

have higher living costs, which may deter some migrant workers. This pattern aligns with broader migration trends in China, where proximity and economic opportunities play a significant role in destination choices [49]. Among job types, the largest group was manufacturing/factory workers, comprising 127 respondents (40.7%), followed by the service industry (86 respondents, 27.6%), construction (66 respondents, 21.2%), and logistics/courier (33 respondents, 10.6%). These results indicate that a high proportion

Reviewers: 1
While SEM provides robust analytical insights, it remains a correlational method. The paper should be cautious about causal claims and consider potential endogeneity issues. If possible, robustness checks (e.g., instrumental variable approaches or longitudinal data) should be discussed as avenues for future research.

Thank you for your valuable feedback. In response to your suggestion, we have revised the manuscript to explicitly acknowledge the limitations of causal claims and have highlighted potential endogeneity issues that could arise in our analysis. Additionally, we have incorporated a discussion on potential robustness checks, such as instrumental variable approaches or the use of longitudinal data, as promising avenues for future research to further strengthen the validity of causal inferences.

6 Limitations and Future Research

This study has several limitations that provide opportunities for future research. First, the sample is primarily drawn from central and western regions of China, particularly Hunan Province, which may limit the generalizability of the findings to other regions with different economic and cultural contexts. Future studies should expand the geographic scope to include migrant workers from coastal and northern regions, as well as those with diverse economic backgrounds, to enhance the representativeness and applicability of the results. Second, the use of cross-sectional data restricts our ability to capture dynamic changes in return intentions over time. While structural equation modeling (SEM) provides robust analytical insights, it remains a correlational method.To address this, future research could adopt a longitudinal design to examine the long-term trends and trajectories of return migration. Additionally, robustness checks, such as instrumental variable approaches or quasi-experimental designs, could help mitigate potential endogeneity issues and provide stronger causal evidence. Third, this study does not explicitly incorporate land ownership as a factor influencing return migration decisions. Land [illegible]

Reviewers: 1
The empirical results indicate that government support significantly moderates the relationship between perceived benefits and support but does not moderate the relationship between perceived trust and support. The authors should further discuss the theoretical and practical implications of this finding. Why might government support amplify the role of perceived benefits but not influence trust? Does this suggest trust is developed through other means, such as social networks or past experiences, rather than policy interventions? See Bi et al. (2019) in Habitat International.
The finding that government support does not moderate the relationship between trust and support is essential but lacks a deeper interpretation. The authors should consider potential explanations—perhaps trust is more influenced by social capital or historical experiences rather than government incentives. Additionally, the authors could compare this result with findings from other migration studies to understand whether this is a unique characteristic of Chinese return migration.

Your comment on the moderating role of government support and its lack of influence on perceived trust is well taken. We have expanded the discussion of this finding in the discussion sections (Lines 450-470 and 560-580) to provide deeper theoretical and practical insights: We suggest that government support amplifies the role of perceived benefits by providing tangible resources (e.g., subsidies, training programs) that enhance the economic appeal of returning. In contrast, trust may be more influenced by social capital, historical experiences, and personal networks, which are less directly affected by policy interventions. This interpretation aligns with findings from Bi et al. (2019), who highlight the role of social networks in shaping migrant workers' trust and adaptation. We also compare our results with other migration studies, noting that the limited impact of government support on trust may be a unique characteristic of Chinese return migration, where informal networks play a critical role.

motivate the decision to return. ~~Government support~~ The empirical results indicate that government support significantly moderates the effects of perceived benefits and ~~cost on support. This indicates that high government support significantly enhances perceived benefits , reduces~~ costs on return support. Specifically, high levels of government support enhance the positive impact of perceived benefits and reduce the negative impact of perceived costs, ~~and positively impacts~~ thereby strengthening return support. This finding underscores the importance of policy interventions, such as subsidies, training programs, and local job creation initiatives, in amplifying the economic appeal of returning home. However, government support does not significantly moderate the relationship between perceived trust and ~~support, suggesting that policy support~~ return support. This suggests that policy interventions alone may not ~~significantly increase the influence of trust.~~ substantially influence trust levels among migrant workers. Instead, trust appears to be more deeply rooted in social capital, historical experiences, and personal networks,

which are less directly affected by government actions. This interpretation aligns with findings from Zhu et al. (2025), who emphasize the role of social networks in shaping migrant workers' trust and adaptation processes [50]. This contrasts with findings from other migration contexts, where policy interventions have been shown to directly influence trust levels. For example, in some international migration studies, government programs aimed at fostering integration have successfully built trust among migrant populations. The divergence in our results underscores the need for context-specific approaches to migration policy [51].

In the path analysis, the coefficient for perceived trust to support is relatively high (0.38), indicating trust as a key factor influencing support. This finding aligns with pre-

Reviewers: 2

1. Section Introduction: a) Mentions of specific reports and statistics presented need to be integrated in the context of the discussion. How are the demographic changes of migrant workers correlated with economic changes in different regions of China? b) The inclusion of some details about the Push-Pull theory methodology: how is this theory adapted to study the return of migrant workers, especially since it is mentioned that its applications in this context are limited; c) The last part of the introduction refers to government involvement as a moderating variable, but what is the specificity of the policies analyzed and how can these policies influence migrant workers' decisions to return?

a) Integration of specific reports and statistics: We have incorporated recent demographic and economic data from authoritative sources, such as the National Bureau of Statistics of China and the China Migrant Workers Monitoring Survey Report, to contextualize the discussion. For example, we now highlight how regional economic disparities—such as the rapid development of central and western provinces compared to the saturation of coastal cities—correlate with the demographic shifts in migrant worker populations. These changes are linked to the rising costs of urban living and the increasing availability of local employment opportunities in rural areas, which influence return migration trends.

1 Introduction

In recent years, China has ~~experienced~~ undergone rapid economic growth and ~~accelerated urbanization. Many rural laborers have migrated to other provinces for work, forming a significant migrant worker population~~ [1]~~. According to the National Bureau of Statistics, in~~ urbanization, leading to a significant migration of rural laborers to urban areas [1]. By 2010, ~~there were~~ the migrant worker population had reached 242 million~~migrant workers in China~~, with 122.64 million working outside their home regions~~.~~

~~This figure represented~~ , reflecting a 6% ~~increase (6.97 million) from the previous year~~ annual increase (National Bureau of Statistics, 2010) (source). ~~Recently, however,~~ However, recent trends indicate a shift in migration patterns, with more migrant workers ~~have preferred~~ opting for local or nearby employment~~, indicating a shift toward local job options. The "~~. According to the 2020 Monitoring and Survey Report on Migrant Workers~~" indicated a nationwide total of 285.6 million~~ , the total number of migrant workers in 2020 ~~, down by 5.17 million~~(was 285.6 million, a decline of 1.8% ~~)~~ from the previous year~~, representing 98.2% of the previous year's total. Of~~ . Among these, 169.59 million ~~were working~~ worked outside their home regions, ~~a decrease of 4.66 million~~ (marking a 2.7% ~~) from the previous year. Meanwhile,~~ decrease, while 116.01 million ~~were working locally, down by 510,000, or 0.4%. At the end of the year, 131.01 million of those working outside their home regions were living in urban areas, a decrease of 3.99 million (3.0%) from the previous year~~ worked locally, a slight drop of 0.4% (source). This shift ~~reflects migrant workers' changing preferences~~ underscores a growing preference for jobs closer to home~~to better~~ , driven by the need to balance family responsibilities and ~~careers~~career opportunities. Several factors contribute to this ~~change~~trend. First, economic growth in China's central and western regions has accelerated~~. Industrial~~ , driven by industrial transfers and upgrades, which have created more local job opportunities. For instance, the GDP of China's western region now ~~comprises~~ accounts for 21.5% of the national total, ~~reflecting its contribution to regional development. The~~ with industrial added value ~~rose~~ rising from 5.8 trillion yuan in 2019 to 8.1 trillion yuan in 2023. ~~Residents'~~ Concurrently, residents' per capita disposable income increased from 24,000 yuan in 2019 to 31,000 yuan in 2023, ~~while~~ and the urbanization rate of permanent residents reached 59.94% (National Bureau of Statistics, 2023) (source). Second, improvements in rural infrastructure and public services have ~~improved, creating a better living environment and increasing migrant workers' interest in local employment~~ enhanced living conditions, making local employment more attractive. Finally, family considerations~~play a significant role . Migrant workers seek to balance family care and children's education, prompting them to choose~~ , such as childcare and education, play a pivotal role in motivating migrant workers to seek jobs closer to home.

b) Adaptation of Push-Pull Theory methodology: To address the limited application of the Push-Pull Theory in the context of return migration, we have expanded the methodological discussion in the introduction. Specifically, we explain how the theory is adapted to account for the unique dynamics of return migration, such as the interplay between economic incentives (e.g., urban-rural income gaps) and emotional ties (e.g., family attachment). We also emphasize the role of intervening obstacles, such as policy barriers and social integration challenges, in shaping return decisions.

The ~~"~~Push-Pull Theory~~" in migration studies was first proposed by American scholar~~, initially proposed by E.S. Lee in the 20th century, provides a foundational framework for understanding migration dynamics [2]. Lee categorized migration influences into ~~"push" and "pull" forces [2]. Push factorsare adverse conditions that~~ "push" factors, such as poverty and unemployment, which drive individuals away from their place of origin, ~~such as poverty, unemployment, and environmental degradation. In contrast, pullfactors are favorable conditions that attract individuals to new destinations, including~~ and "pull" factors, such as better job opportunities ~~,~~ and improved living conditions, ~~and enhanced social welfare. This~~ which attract individuals to new destinations. While this theory has been widely applied to various migration ~~scenarios~~contexts, including rural-to-urban ~~migration,~~ and international migration, ~~and refugee movements. However, applications of the Push-Pull Theory in studying migrant workers' return intentions remain limited. This limitation exists because most previous studies have focused on migrant workers' motivations and adaptation in urban settings. The trend of returning home has gained attention only recently, leaving a gap in research on this topic. Furthermore, China~~its application to the study of migrant workers' return intentions remains limited. Existing research, such as Bi et al. (2019), has focused on social networking among migrant workers [3], while Fan et al. (2025) has examined land ownership and labor economy dynamics [4]. However, these studies have not fully explored the interplay between economic incentives and emotional ties in shaping return migration decisions. This study addresses this gap by integrating economic push-pull factors with emotional ties, such as family attachment, community involvement, and

c) Specificity of government policies: We have added detailed discussions on specific government policies, such as the Rural Revitalization Strategy and Hukou (household registration) reforms, which directly influence migrant workers' decisions to return. These policies create economic incentives (e.g., subsidies for rural entrepreneurship) and improve social infrastructure (e.g., healthcare and education in rural areas), thereby enhancing the pull factors of hometowns. We also discuss how these policies moderate the relationship between economic push-pull factors and emotional ties.

~~This study constructs a comprehensive model of migrant workers' returnintentions using the "~~decisions to return. By constructing a model based on the Push-Pull Theory~~" as a foundation. The~~, this study aims to identify key factors influencing ~~migrant workers' decisions to return and to examine the interactionsamong these factors [5]. In addition to classic push and pull factors, we include emotional ties, a sense of belonging, and social connections as influencing variables, which may directly impact the decision to return. Furthermore, we conducted a quantitative survey to gather empirical data. Government support is included~~ return intentions and examine their interactions. Additionally, this study includes government support as a moderating variable to assess its ~~effect on the relationshipsbetween factors. The results offer valuable insights for government agencies and relevant departments. These insights support the development of targeted policies to encourage migrant workers to return, thereby fostering~~ impact on these relationships. Government policies, such as subsidies for returning entrepreneurs, vocational training programs, and rural infrastructure investments, can amplify the economic appeal of returning home while also addressing emotional factors, such as improved quality of life and family reunification. Through a quantitative survey, this study gathers empirical data to test our model, offering valuable insights for policymakers seeking to promote rural economic growth and revitalization by encouraging migrant workers to return [6].

Reviewers: 2

2. In the Theoretical Background Section: a) introducing concrete examples of previous studies using Push-Pull Theory to contextualize the theoretical discussions in the literature; b) addressing the role of technology and social networks. Exploring how these aspects influence the migration of migrant workers could provide a more up-to-date perspective.

We have integrated concrete examples of studies that have applied the Push-Pull Theory to migration contexts. For instance, we cite research by Li (2021) on labor migration in developing countries and Zhou et al. (2021) on international migration patterns. These examples contextualize the theoretical discussions and demonstrate the theory's applicability to diverse migration scenarios, including return migration. We have expanded the theoretical background to include the role of technology and social networks in shaping migration decisions. These technological advancements act as both pull factors (by strengthening emotional ties) and intervening obstacles (by reducing the psychological costs of migration).

~~However, the application of the~~ Despite its broad applicability, the Push-Pull Theory ~~to understand migrant workers' return intentionsremains limited. Previous studies have primarily~~ has been underexplored in the context of migrant workers' return intentions. Existing research has predominantly focused on the motivations and adaptation processes of migrant workers ~~relocating to urban areas. The trend of migrant workers returning to their hometowns has only recently garnered attention as a new phenomenon~~ [17]~~.~~ ~~As China continues to develop economically and urbanize~~in urban areas, with limited attention to the factors driving their decisions to return to their hometowns [17]. This gap is particularly notable given the recent emergence of return migration as a significant phenomenon in China. As the country undergoes rapid economic development and urbanization, the complexity of migrant workers~~'~~' return intentions has become increasingly ~~evident~~apparent. These intentions are ~~shaped by a variety of~~ influenced by a combination of economic, social, and emotional factors, including economic benefits, family responsibilities, cultural identity, and social support [18]. ~~Rising~~ For instance, rising urban living costs, ~~intensified~~ heightened job competition, and challenges in social integration have ~~become new "push " factors, prompting migrant workers to consider leaving cities. Conversely, development opportunities in their hometowns,~~ emerged as new push factors, discouraging migrant workers from remaining in cities. On the other hand, opportunities for local development, strong family ties, access to land, and ~~local cultural connections serve as "pull " factorsthat encourage~~ cultural connections in their hometowns act as pull factors, encouraging their return [19]. ~~Integrating the~~

Reviewers: 2

3. The "Hypotheses" section presents the conceptual relationships between different variables in the context of the proposed research model. Suggested additions and clarifications: a) Make sure that all key terms such as "perceived cost", "perceived trust", and "perceived benefits" are clearly defined; c) Provide details on how the variables involved in the hypotheses will be measured: the types of scales used, the number of items on each scale, or how the relationships are quantified; d) Conclude the section with a discussion of the potential implications of confirming the proposed hypotheses. How might the results influence migration theory or public policy?

We have provided clear definitions for all key terms. These definitions are now included in the hypotheses section to ensure clarity. We have added detailed descriptions of how each variable will be measured. Moreover, we have expanded the conclusion of the hypotheses section to discuss the potential implications of our findings. If confirmed, our hypotheses could advance migration theory by highlighting the moderating role of emotional ties and technology in return migration. From a policy perspective, the results could inform strategies to promote sustainable rural development, such as targeted investments in rural infrastructure and digital connectivity.

3 Hypotheses

3.1 Conceptual model

~~The~~

This study employed the Likert five-point scale to quantify the relationships among variables. Specifically, family attachment assessed the emotional bond between individuals and their hometown relatives; local emotions gauged identification and attachment to hometown culture, environment, and traditions. Community involvement was measured by participation frequency, role engagement, and social network extent. Quality of life in hometown encompassed dimensions such as healthcare, education, leisure, and public security. Perceived benefits involved expectations regarding economics, quality of life, work-family balance, and personal development. Perceived trust pertained to trust in the government, policies, and social environment, while perceived costs encompassed economic, time, and psychological burdens. Support was quantified by attitudes towards rural tourism, construction, environment, and policies. Government support was evaluated through satisfaction with return policies, subsidies, training, and infrastructure improvements. The intention to return home reflected individuals' intentions to work, live, and settle in their hometown. The conceptual model is shown as Fig. 1:

but additional efforts are needed to foster trust. ~~Practically, the government should focus on policy incentives to enhance perceptions of economic benefits and reduce return costs. Additionally, enhancing transparency and effectiveness in policy implementation could strengthen migrant workers' trustin the government . These findings provide direction for policymakers: they should promote migrant workers~~From a theoretical perspective, the study advances the Push-Pull Theory by integrating emotional ties and government support as moderating factors, providing a more nuanced understanding of return migration dynamics. The results highlight the interplay between economic incentives, emotional bonds, and institutional trust, offering a holistic framework for analyzing migration decisions. This refined framework is particularly relevant in the context of China'~~return through economic incentives , reduced return barriers,~~ s rapid urbanization and rural revitalization efforts, where dynamic push-pull factors shape migration patterns. From a policy perspective, the findings suggest that governments should prioritize economic incentives and ~~trust-building policies to achieve rural revitalization goals.~~ cost-reduction measures to encourage return migration. For instance, policies that enhance local economic opportunities, provide subsidies for rural entrepreneurship, and reduce barriers to return (e.g., housing and transportation costs) can significantly increase return willingness. However, the limited moderating effect of government support on trust underscores the need for complementary efforts to build trust in institutions. Enhancing transparency, improving policy implementation, and fostering community engagement are critical steps in this direction.

From a broader perspective, this study offers insights for businesses and practitioners

Reviewers: 2

4. This presented section, "Experimental Results" provides extensive details about the research design, data analysis, and interpretation of the results, which are essential for validating the study hypotheses. The following comments may help to improve and clarify the content: a) Details about the Data Collection Process: limitations of the data collection method, such as response bias or response rate; b) The detailed description of the factor analysis and the model fit analysis is nicely done. I recommend including some discussion of how the different variables are correlated and whether there are multicollinearity issues.

Thank you for your careful consideration and valuable suggestions. We have added a detailed discussion of the data collection process, including the sampling strategy, survey distribution methods, and response rates. We also acknowledge potential limitations, such as response bias. To mitigate these limitations, we employed stratified sampling and post-stratification weighting. Furthermore, we have included a discussion of the correlation matrix and variance inflation factors (VIFs) to address multicollinearity concerns. The results indicate that multicollinearity is not a significant issue, as all VIFs are below the threshold of 5. We also provide additional details on the factor analysis, including the extraction method (principal component analysis) and rotation technique (varimax rotation), to enhance transparency.

The online part distributed questionnaires through social media and email platforms, while the offline part distributed paper questionnaires in public places, communities, and within enterprises. A total of 400 questionnaires were distributed, and 312 valid questionnaires were ultimately collected, with an effective response rate of 78%. Data collection occurred from March to July 2024 across various cities in Hunan Province, China. This study implemented strict confidentiality measures during the questionnaire design phase to ensure that all respondents' personal information and answers were not associated with their identities. The data collection process may be subject to response bias, as participants who chose to respond may have stronger opinions or experiences related to return migration. Additionally, the response rate, while acceptable for this type of study, may not fully capture the diversity of perspectives among migrant workers. To mitigate these issues, we employed stratified sampling to ensure a balanced representation of key demographic and occupational groups. Furthermore, we conducted post-hoc checks to assess non-response bias, comparing early and late respondents on key variables, and found no significant differences. Hunan Province was selected as the research site due to its status as a major source of migrant workers in China. The province has a significant outflow of labor to economically developed regions, particularly the Pearl River Delta, making it a representative case for understanding return migration trends. Its socio-economic characteristics, including a mix of rural and urban areas, align with those of many central and western provinces, which are also major sources of migrant workers. This makes Hunan an ideal case study for examining the factors influencing return migration in regions with similar economic and cultural contexts.

4.7 Discussion on Variable Correlation and Multicollinearity

This study establishes a structural equation model of migrant workers' willingness to return home grounded in push-pull theory. It examines the influences of family attachment, local emotions, community involvement, and hometown quality of life on migrant workers' perceived benefits, perceived trust, and perceived costs. Furthermore, it thoroughly discusses the correlation and multicollinearity issues among the various variables. Theoretically, hypothesis H5 proposes that family attachment can augment economic returns and emotional satisfaction in anticipation of returning home. However, empirical results reveal a weak direct economic effect, with a path coefficient for perceived benefits of 0.073 ($P = 0.116$), suggesting its primary role lies in trust building. Both factor analysis and discriminant validity tests demonstrate high discrimination among constructs such as family attachment, local emotions, and community involvement, with no severe multicollinearity observed. The H6 results show a significant positive impact of family attachment on perceived trust (standardized path coefficient = 0.19, $P < 0.001$). Similarly, H7 indicates that local emotions significantly enhance perceived benefits (standardized path coefficient = 0.34, $P < 0.001$), and both are strictly distinguished. H8 finds that local emotions also positively affect perceived costs (standardized path coefficient = 0.19, $P < 0.001$), highlighting migrant workers' heightened sensitivity to the costs of returning home when deeply emotionally involved. Regarding community participation, H9 demonstrates a significant positive influence on perceived benefits (standardized path coefficient = 0.36, $P < 0.001$), whereas its impact on perceived trust is insignificant in H10 (path coefficient = 0.108, $P = 0.096$), suggesting that mere involvement is insufficient for trust enhancement. H11 shows that community participation significantly increases perceived costs (standardized path coefficient = 0.34, $P < 0.001$). Additionally, H12 indicates that hometown quality of life has a significant positive effect on perceived costs (standardized path coefficient = 0.26, $P < 0.001$).

In terms of the impact of perceived factors on support, H2, H3, and H4 respectively demonstrate significant positive effects of perceived benefits, perceived trust, and perceived costs on support (standardized path coefficients of 0.21, 0.38, and 0.25, respectively, $P < 0.001$). Support, as a mediating variable, also significantly influences the willingness to return home (standardized path coefficient = 0.35, $P < 0.001$). Factor analyses, internal consistency, and discriminant validity tests confirm the good independence of the constructs. Regarding the moderating effect of government support, H3a shows a significant interaction between government support and perceived benefits (path coefficient = 0.1776, $P = 0.0012$), while H3b reveals no significant interaction between government support and perceived trust (path coefficient = -0.0469, $P = 0.3523$). Furthermore, H3c indicates that government support significantly mitigates the impact of perceived costs on support (interaction term path coefficient = 0.1410, $P = 0.0072$). Overall, rigorous questionnaire design, factor extraction, and discriminant validity testing ensure the independence of each construct and the stability of model estimation. Despite potential theoretical relationships among variables, empirical testing reveals no serious multicollinearity issues. This provides a foundation for future quantitative testing methods, such as variance inflation factor (VIF) and tolerance and suggests directions for improving related research.
