# Supplementary material for: Why do Chinese migrant workers return? Exploring economic push-pull factors and emotional ties
Source: PLoS One. 2025 Jul 17;20(7):e0325848. doi: 10.1371/journal.pone.0325848 (PMC12270181; doi:10.1371/journal.pone.0325848)
Supplement: Attachment — Submitted filename: Response to Reviewers-14.pdf [file pone.0325848.s004.pdf]

Dear Editor and Reviewers,

Thank you for your constructive feedback on our manuscript. We sincerely appreciate the time and effort you have dedicated to reviewing our work. We have carefully considered all the comments and have revised the manuscript accordingly. Below, we provide a point-by-point response to the reviewers’ suggestions.

Response to Reviewer #1:

We are grateful for your positive evaluation of our paper and your insightful suggestions for improvement. Below, we address your minor comments in detail:

> 1) The manuscript claims that living costs in Beijing and Shanghai are higher than in Guangzhou and Shenzhen. To strengthen this assertion, the authors should provide supporting statistical evidence, such as cost-of-living indices, rent data, or other economic indicators.

As suggested, we have added supporting statistical evidence to strengthen our claim. We incorporated cost-of-living indices and rent data from the National Bureau of Statistics of China (2023) and city-specific consumer price reports.

> and service sectors. In contrast, Beijing, and Shanghai, while economically dominant, are farther away and have higher living costs, which may deter some migrant workers. According to the 2023 China Statistical Yearbook, the average monthly rent per square meter in Beijing (¥86.5) and Shanghai (¥82.1) was approximately 30% higher than in Guangzhou (¥62.3) and Shenzhen (¥65.8). Additionally, the ECA International 2022 Cost of Living Survey ranked Beijing and Shanghai among the top 10 most expensive
>
> 10/27
>
> Chinese cities, while Guangzhou and Shenzhen fell outside the top 15 [53].This pat-

2) The study's extension of the Push-Pull Theory to include emotional bonds is well-argued. A recent study by Lee et al. (2025) on Chinese migrant settlements in Greater Sydney offers empirical support for the role of social networks in shaping location preferences. Specifically, Chinese migrants prefer areas with larger Chinese communities, as these provide social engagement opportunities and facilitate settlement. The authors may consider referencing the following study to support this point in the theoretical background.
https://doi.org/10.1016/j.habitatint.2025.103331

Thank you for your suggestion. We have integrated the suggested study by Lee et al. (2025) into our theoretical framework (Literature Review section). This reference strengthens our discussion on how emotional bonds (e.g., community ties) influence migration decisions.

2.2 The "Push-Pull Theory" in Migration Theory

The Push-Pull Theory, introduced by Everett S. Lee in the 1960s, serves as a foundational framework in migration studies [11]. This theory categorizes migration drivers into "push" and "pull" factors. Push factors refer to adverse conditions in the place of origin, such as poverty, unemployment, resource scarcity, and environmental degradation, which compel individuals to migrate. Conversely, pull factors represent favorable conditions in the destination, including better job opportunities, improved quality of life, and enhanced social welfare, which attract migrants [12]. Six key factors that drive their location settlement decisions have been identified regarding Chinese settlement preferences. These include educational resources, public transportation, infrastructure and amenities, economic and employment opportunities, environment, and safety [13–15]. Additionally, Lee emphasized the role of intervening obstacles—such as distance, cost, policy restrictions, and information asymmetry—as well as individual characteristics like age, education level, family status, and personality traits, in shaping migration decisions [16]. Over the decades, the Push-Pull Theory has been widely applied to various migration contexts, including rural-to-urban migration, international migration, and disaster-induced displacement [17]. For instance, studies by Kwilinski et al. (2022) and Sanliturk et al. (2023) have utilized the theory to explain labor migration patterns in developing countries, highlighting the interplay between economic disparities and migration flows [18, 19]. According to Hormozinejad et al.'s (2024) research, Chinese immigrants in Sydney consider tangible factors such as educational resources, public transportation, infrastructure, economy, environment, and safety when choosing suburbs, as well as intangible factors such as cultural identity and Chinese cultural atmosphere. The Chinese would choose to settle in an area because of the advantageous characteristics exhibited in the suburb [20].

13. Martinez, B. P. (2021). The case of Cubans: Racial inequality in U.S. Homeownership and home values. *Sociological Forum*, *36*(1), 181–205.

14. MacDonald, H., Galster, G., & Dufty-Jones, R. (2018). The geography of rental housing discrimination, segregation, and social exclusion: New evidence from Sydney. *Journal of Urban Affairs*, *40*(2), 226–245.

15. Lee, C. L., Bangura, M., & Lin, J. (2025). Residential segregation of Chinese minority groups in Greater Sydney. *Habitat International*,158, 103331.

We believe these revisions have enhanced the rigor and clarity of our manuscript. Thank you again for your valuable input. Please find our revised manuscript attached, with all changes highlighted for ease of review.

Sincerely,

Zhou Qiong
Hunan Agricultural University, Hunan, China
