# Supplementary material for: Why do Chinese migrant workers return? Exploring economic push-pull factors and emotional ties
Source: PLoS One. 2025 Jul 17;20(7):e0325848. doi: 10.1371/journal.pone.0325848 (PMC12270181; doi:10.1371/journal.pone.0325848)
Supplement: Attachment — Submitted filename: Response to Reviewers_5.15.docx [file pone.0325848.s005.docx]

Dear Reviewer,

We sincerely appreciate your time and constructive feedback on our manuscript. Your comments have significantly helped us improve the clarity, rigor, and coherence of the paper. This study aims to systematically investigate the factors driving the increasing trend of return migration among Chinese migrant workers by integrating economic and emotional perspectives within the framework of Push-Pull Theory. The study seeks to advance migration theory by developing a comprehensive model that bridges the gap between economic and emotional determinants of return migration, while also providing practical insights for rural revitalization policies by demonstrating that effective strategies must address both material incentives and trust-building measures. Below, we address each of your suggestions in detail and outline the revisions made in response. We believe these revisions have strengthened the manuscript significantly. Thank you again for your valuable input. Please let us know if further adjustments are needed.

Sincerely,

Zhou Qiong

Hunan Agricultural University, Hunan, China

**Response to comments：**

Reviewer #3:

1.The construct of "support" is central to the analysis but remains ambiguously defined at some points. Please clearly define "support" early in the conceptual framework and maintain a consistent interpretation throughout the paper.

We agree that it is necessary to provide a clearer definition of 'support'. In the revised manuscript, our conceptual framework (Section 3.2-3.3) explicitly defines "support" as the attitude of migrant workers towards returning home to develop their careers. We also ensured consistency in its use throughout the entire paper, particularly in the Methods and Discussion sections.

Reviewer #3:

2.The representativeness of the sample is questionable. The manuscript does not clearly explain how respondents were selected or whether a random sampling method was used. Please clarify the sample selection procedure and discuss potential biases arising from non-random sampling.

We greatly appreciate your careful attention to the reference details. We acknowledge this limitation and have now provided a detailed explanation of our sampling strategy in Section 3.1 (Data Collection).

Reviewer #3:

3.Return migration decisions are influenced by both destination and origin factors. Since the study’s sample is limited to Hunan Province, regional variations are not considered, which may affect the generalizability of the findings. Please explicitly acknowledge this limitation and discuss how regional differences could impact return migration behavior.

We have expanded the Limitations section (Section 6.2) to explicitly address this issue. Key additions include: Acknowledging that regional economic disparities (e.g., coastal vs. inland provinces) may influence migration decisions differently. Recommending future studies to incorporate multi-regional samples for broader generalizability.

Reviewer #3:

4.Some hypotheses (e.g., H5 and H10) were not supported, but no interpretation or explanation is provided. Please briefly discuss plausible reasons for these nonsignificant results in the discussion section.

We sincerely appreciate your valuable suggestions. We have added a dedicated paragraph in the Discussion (Section 4.8) to interpret nonsignificant findings:

Reviewer #3:

5.The manuscript lists too many keywords. Please limit the keywords to 4–6 to align with common academic publishing standards and enhance focus.

We sincerely thank you for your valuable suggestion. We have reduced the keywords to the following six core terms.

Reviewer #3:

6.The manuscript contains too many subdivisions, resulting in fragmented sections and occasional disruptions to the logical flow. Please consider simplifying the chapter structure by merging related sections and reducing the number of headings to improve coherence and readability.

We deeply appreciate your positive feedback and encouragement. We have tried to delete some sections as much as possible to improve readability.
